# Fatigue Life Prediction for Transverse Crack Initiation of CFRP Cross-Ply and Quasi-Isotropic Laminates

**DOI:** 10.3390/ma11071182

**Published:** 2018-07-10

**Authors:** Atsushi Hosoi, Hiroyuki Kawada

**Affiliations:** 1 Department of Applied Mechanics and Aerospace Engineering, Waseda University, Tokyo 169-8555, Japan; kawada@waseda.jp; 2 Kagami Memorial Research Institute for Material Science and Technology, Waseda University, Tokyo 169-0051, Japan

**Keywords:** carbon fiber reinforced plastic laminates, transverse crack, fatigue, initiation, prediction

## Abstract

Carbon fiber reinforced plastic (CFRP) laminates are used as main structural members in many applications. Transverse cracks that form in 90° layers of CFRP laminates are mostly initial damage in the case where tensile loading is vertically applied to the 90° layers of CFRP laminates, and they are the origin of more serious damage of delamination and fiber breakage. It is thus important to predict quantitatively the transverse crack initiation of CFRP laminates subjected to cyclic loading to ensure the long-term reliability of the laminates. The initiation and multiplication behaviors of transverse cracks strongly depend on the laminate configuration, thickness, and thermal residual stress. Therefore, a model based on the Walker model was proposed to predict transverse crack initiation in CFRP cross-ply and quasi-isotropic laminates under cyclic loading in the present study. The usefulness of the proposed model was verified with 10 different CFRP laminates formed from four different prepregs with epoxy resin matrices. The analysis results were in good agreement with experimental results. The fatigue life was expressed with three constants, which related to the fatigue strength reduction, the normalized fatigue strength at *N* = 1 cycle, and the contribution of stress amplitude to the fatigue life, and they are independent of the laminate configuration.

## 1. Introduction

Carbon fiber reinforced plastic (CFRP) laminates are used as the main structural members of aircraft, automobiles, wind turbine blades, tidal turbine blades, and jet-engine fan blades. The damage tolerance strain of CFRP laminates in the design of airplanes is determined based on the compression-after-impact strength. However, the safe-life design should be considered for more precise design and long-term durability of CFRP structures in other applications, such as automobiles in various environments, where the structures are not necessarily inspected and repaired in controlled operations, like those implemented for airplanes. The main forms of the damage mechanism of CFRP laminates are fiber-matrix debonding, intralaminar and interlaminar matrix cracks, and fiber breakage, which propagate while interacting with each other, finally, resulting in the rupture of the laminates [1], and many fatigue life prediction models, which are based on cumulative damage theory [2], strain energy density [3], statistical approach [4], constant fatigue diagram [5], and damage mechanics [6], have been proposed so far. A transverse crack in 90° layers of CFRP laminate is mostly initial damage and is the origin of more serious damage of delamination and fiber breakage. It is thus essential to evaluate quantitatively the fatigue life to transverse crack initiation of CFRP laminates for the use of the laminates as structural members.

The multiplication and propagation behaviors of transverse cracks in CFRP laminates have been quantitatively evaluated through experiments and analysis under static and fatigue loading [7,8,9,10,11,12]. The initiation behavior of transverse cracks under static loading has also been evaluated [7,8,13,14,15,16]. Recently, the effects of initial defects on crack initiation were evaluated by Maragoni et al. [17] and Aratama et al. [18]. However, there have been few studies on the prediction of transverse crack initiation in CFRP laminates under fatigue loading because it is difficult to experimentally or analytically evaluate the initiation behavior of a transverse crack under fatigue loading. Henaff-Gardin and Lafarie-Frenot [19] experimentally showed that the fatigue life to transverse crack initiation can be represented by a power law function of the normalized energy release rate, the maximum energy release rate of fatigue loading for the critical energy release rate, associated with the transverse crack formation. Ogi and Yashiro [20] proposed a probabilistic slow crack growth model with which to predict the initiation and multiplication behavior of transverse cracks in CFRP laminates under fatigue loading. Quaresimin et al. [21,22] evaluated the fatigue crack initiation in unidirectional and off-axis laminates with local hydrostatic stress or local maximum principal stress. Recently, the authors proposed a method of predicting the fatigue life to transverse crack initiation by applying a power law function that expresses the relationship between the transverse crack multiplication rate and the energy release rate range associated with transverse crack formation [23]. In addition, we showed quantitatively that the fatigue life to transverse crack initiation in cross-ply laminates can be predicted using the fatigue properties obtained from the S–N curve of a unidirectional laminate in transverse direction, 90° unidirectional laminate. [24,25].

The initiation and multiplication of the transverse cracks in CFRP laminates strongly depend on the stacking sequence of laminates, dimensions, thermal residual stress, and the stress ratio. In some cases, transverse cracks pass through the width direction of the laminates; however, in other cases, short transverse cracks multiply at the edges of the laminates because of free-edge effects. As described above, a model that can be used to evaluate the initiation of transverse cracks that vary under such effects in the case of fatigue loading has not yet been proposed. Therefore, the present paper proposes a model based on fracture mechanics with which to evaluate the fatigue life to transverse crack initiation considering these effects and verifies the usefulness of the model using various types of cross-ply [0*_m_*/90*_n_*]_s_ and quasi-isotropic [45/0/–45/90]_s_ laminates.

## 2. Prediction Model for Transverse Crack Initiation

The present paper proposes a model with which to predict transverse crack initiation in cross-ply and quasi-isotropic laminates with the application of cyclic loading. A thermal residual stress is generated in the laminates because of the difference in the thermal expansion coefficients of the plies and the difference between the test and curing temperatures. A tensile thermal residual stress is thus generated in the 90° layers of the laminates at room temperature. Therefore, even if mechanical loading with a constant stress ratio of *R* = 0.1 was applied to the specimens, the stress ratio applied to the 90° layers of the laminates differed depending on the laminate configuration and the applied stress level resulting from the thermal residual stress. In addition, the stress at the transverse crack initiation was affected by a size effect [26]. To evaluate the transverse crack initiation, the effects of the stress ratio and laminate thicknesses must be considered.

Models, such as the Gerber model, Goodman model, and Soderberg model, have been proposed to evaluate the effect of the stress ratio on the prediction of the fatigue life [27]. Walker [28] proposed an equation with which to evaluate the effect of the stress ratio on fatigue crack propagation in metallic materials:

(1) $$\frac{da}{dN}=A{\left(K_{\mathrm{eq}}\right)}^{m},$$

(2) $$K_{\mathrm{eq}}=K_{\max}^{1-\gamma }\Delta K^{\gamma },$$

where *a* is the crack length, *N* is the number of loading cycles, and *K* is the stress intensity factor. Δ*K* is the range of the stress intensity factor (Δ*K* = *K*_max_ − *K*_min_), while *A*, *m,* and *γ* are constants obtained from experimental results. Because the growth of a fatigue crack is controlled by *K*_max_ in brittle materials and by Δ*K* in ductile materials [29], fatigue crack growth in many materials is generally controlled by both *K*_max_ and Δ*K*, as shown in Equation (2). Hojo et al. [30,31] evaluated the effect of the stress ratio on mode-I and mode-II delamination growth in CFRP laminates using the energy release rate. It is more common to use the energy release rate, *G*, because a CFRP is an inhomogeneous material composed of fibers and a matrix resin. In linear fracture mechanics, the relationship between the energy release rate and stress intensity factor is expressed as *G* = *HK*^2^, where *H* is a constant. Equation (2) can thus be expressed according to the strain energy:

(3) $$G_{\mathrm{eq}}={\left({\sqrt{G_{\max}}}^{1-\gamma }\Delta {\sqrt{G}}^{\gamma }\right)}^{2},$$

(4) $$\Delta \sqrt{G}=\sqrt{G_{\max}}-\sqrt{G_{\min}}.$$

Here, the transverse crack multiplication behavior in CFRP laminates under fatigue loading is expressed by:

(5) $$\frac{dD}{dN}=F{\left(\frac{\Delta G_{\mathrm{teq}}}{G_{\mathrm{ti}}}\right)}^{\beta },$$

where *D* is the transverse crack density, which is defined as the number of transverse cracks, *n*, per gauge length, *L* (=*n*/*L*). *F* and *β* are constants obtained in experiments. *G*_teq_ is the equivalent energy release rate associated with the transverse crack formation. The subscript, t, indicates a transverse crack. *G*_ti_ is the energy release rate when the transverse crack initiates under static tensile loading. The energy release rate associated with the transverse crack formed in CFRP laminates is calculated using the model proposed by Nairn [8,14] as:

(6) $$\begin{array}{l} G_{t}={\left(\frac{E_{A}^{\left(90\right)}}{E_{0}}\sigma_{0}-\frac{bE_{A}^{\left(S\right)}E_{A}^{\left(90\right)}}{hE_{0}}\Delta \alpha T\right)}^{2}C_{3}bY\left(D\right)\\ \;={\left(\sigma^{\left(90\right)}\right)}^{2}C_{3}bY\left(D\right) \end{array}.$$

Considering the thermal residual stress, the stress applied on the 90° layers in the laminates is expressed using the law of mixtures:

(7) $$\sigma^{\left(90\right)}=\frac{E_{A}^{\left(90\right)}}{E_{0}}\sigma_{0}-\frac{bE_{A}^{\left(S\right)}E_{A}^{\left(90\right)}}{hE_{0}}\Delta \alpha T=E_{A}^{\left(90\right)}\varepsilon_{0}-\frac{bE_{A}^{\left(S\right)}E_{A}^{\left(90\right)}}{hE_{0}}\Delta \alpha T,$$

where the superscripts (90) and (S) respectively represent 90° layers and the sublaminate adjacent to the 90° layers. *E*_0_ and *E*_A_ are the Young’s moduli of the composite in the axial direction of the 90° laminate and sublaminate, respectively; *σ*_0_ and *ε*_0_ are the stress and strain in the composites, respectively; Δ*α* is the difference in the thermal expansion coefficient between the transverse and longitudinal directions of unidirectional laminates, Δ*α* = *α*_T_ − *α*_A_; *T* is the difference between the specimen temperature and stress-free temperatures; *C*_3_ is a constant that depends on the material properties and ply thickness; *b* and *h* are the one-sided thickness of the sublaminate and half the thickness of the cross-ply or quasi-isotropic laminates, respectively; and *Y*(*D*) is a function that depends on the transverse crack density.

The first transverse crack formation is asymptotically predicted from the transverse crack multiplication behavior expressed by Equation (5). The number of cycles when the transverse crack density increases from *D* to 2*D* is expressed based on Equation (5) as:

(8) $$N\left(2D\right)-N\left(D\right)=\frac{2D-D}{F}{\left(\frac{G_{\mathrm{teq}}}{G_{\mathrm{ti}}}\right)}^{-\beta }.$$

In the region of low transverse crack density in the early stage of fatigue, the energy release rate associated with transverse crack formation is constant. Experimental evidence has shown that the increase in the transverse crack density is proportional to the number of cycles in this region [24]. When the density in the transverse crack initiation is defined as 1/*L*, such that there is one transverse crack in the gauge length *L*, the number of cycles to transverse crack initiation is equal to the number of cycles that it takes for the transverse crack density to increase from 1/*L* to 2/*L*. Therefore, the number of cycles to transverse crack initiation is obtained by substituting *D* = 1/*L* into Equation (8):

(9) $$N_{i}=N\left(2/L\right)-N\left(1/L\right)=\frac{1}{LF}{\left(\frac{G_{\mathrm{teq}}}{G_{\mathrm{ti}}}\right)}^{-\beta }.$$

Here, the normalized energy release rate at a transverse crack density, *D* = 1/*L*, can be expressed based on Equation (6) as:

(10) $${\frac{G_{\mathrm{teq}}}{G_{\mathrm{ti}}}|}_{D=1/L}={\frac{{\left({\sqrt{G_{\max}}}^{\left(1-\gamma \right)}\Delta {\sqrt{G}}^{\gamma }\right)}^{2}}{G_{\mathrm{ti}}}|}_{D=1/L}={\left(\frac{\sigma_{\max}^{\left(90\right)}{}^{\left(1-\gamma \right)}{\left(2\sigma_{a}^{\left(90\right)}\right)}^{\gamma }}{\sigma_{\mathrm{ti}}^{\left(90\right)}}\right)}^{2}.$$

Here, *σ*_max_, *σ*_a_, and *σ*_ti_ are the maximum stress of the applied cyclic loading, the stress amplitude of the applied cyclic loading, and the initiation stress of the transverse crack under tensile loading, respectively. The normalized energy release rate is expressed using only the stress applied in the 90° layers. The shape function of the laminates is erased by normalizing the energy release rate and can be evaluated regardless of the laminate size. Finally, the transverse crack initiation can be predicted by substituting Equation (10) into Equation (9):

(11) $$N_{i}=\eta {\left(\frac{\sigma_{\max}^{\left(90\right)}{}^{1-\gamma }\sigma_{a}^{\left(90\right)}{}^{\gamma }}{\sigma_{\mathrm{ti}}^{\left(90\right)}}\right)}^{\lambda }.$$

Here, *η* and *λ* are constants obtained in experiments. Further, Equation (11) can be expressed as Equation (12):

(12) $$\log N_{i}=\lambda \log\left(\frac{\sigma_{\max}^{\left(90\right)}{}^{1-\gamma }\sigma_{a}^{\left(90\right)}{}^{\gamma }}{\sigma_{\mathrm{ti}}^{\left(90\right)}}\right)+\log\eta .$$

In terms of a logarithmic graph of the relationship between the normalized stress and the fatigue life to transverse crack initiation, *λ* is the slope and *η* is the intercept of the graph. That is, the physical meanings of *λ* and *η* are the parameters related to the fatigue strength reduction in the transverse crack initiation and the normalized fatigue strength in the transverse crack initiation at *N* = 1 cycle, respectively. In addition, the fatigue properties of the material are generally dominated by maximum stress, *σ*_max_, in the case of brittle materials, and by the stress amplitude, *σ*_a_, in the case of ductile materials. Therefore, *γ* is a parameter indicating the contribution of the stress amplitude on the fatigue life to the transverse crack initiation in the samples. Because Equations (11) and (12) are expressed using only the stress applied in 90° layers, the constants, *η* and *λ*, can be obtained from the S–N curve for the transverse crack initiation of arbitrary laminates or the S–N curve of unidirectional 90° laminates. The fatigue life of transverse crack initiation in cross-ply and quasi-isotropic CFRP laminates can then be predicted. The proposed model is verified by reorganizing data of experiments previously carried out by the authors [24,32,33,34,35,36]. 

## 3. Experiments

### 3.1. Specimens

**Figure 1:**
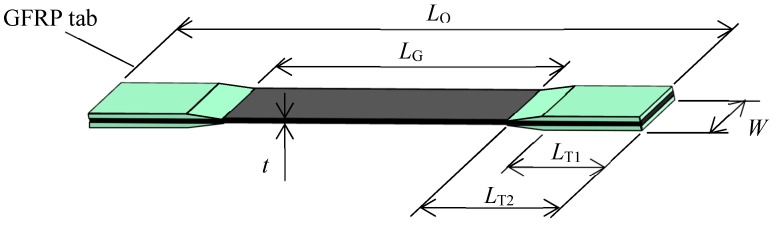
Schematic illustration of the specimen geometry.

Ten CFRP laminates were formed from four prepregs with epoxy resin matrices: T800S/2592 with fiber volume fractions (*V*_f_) of 68% and 61%, T800S/3900-2B with *V*_f_ = 56%, and T800H/3631 with *V*_f_ = 57%. The T800S/3900-2B prepreg was coated with a toughened layer. The cured ply thickness was approximately 80 μm, 110 μm, 190 μm, and 140 μm, respectively. The specimens were cut out from 300 mm × 300 mm laminates using a diamond saw. Figure 1 is a schematic illustration of a specimen, while Table 1 lists the stacking sequences of the laminates and the curing temperatures and dimensions of the specimens. The mechanical properties of each prepreg are listed in Table 2. The edge surfaces of the specimens were polished with emery paper and finished by buffing with diamond powder with particle sizes less than 6 μm. After polishing the specimens, it was confirmed that transverse cracks were not generated with an optical microscope before the tests.

### 3.2. Static Tensile Tests

The mechanical properties and the stress of the transverse crack initiation, *σ*_ti_, of the specimens were obtained in static tensile tests. The tensile speed was 0.5 mm/min. The damage to the specimen was examined by ex-situ observation employing soft X-ray photography (Softex Corp., Tokyo, Japan) and optical microscopy (Olympus Corp., Tokyo, Japan). For soft X-ray photography, a contrast agent of zinc iodide was applied from the edge surfaces of the specimens. At this time, a load sufficiently smaller than that causing the transverse crack was applied to the specimen so that the contrast agent easily penetrated the specimen. The irradiation conditions for soft X-ray photography differ depending on the thickness of the specimen. For example, when observing the specimen with the thickness of 1 mm, the irradiation condition was a voltage of 40 kV, a current of 1 mA, and an irradiation time of 15 s. The developed X-ray photographs were scanned to a computer, and the initiation and growth of the damage was observed. The specimen was removed from the testing machine to observe the damage, and the transverse crack initiation was evaluated by applying a tensile load step by step. Until a transverse crack initiated, the load was applied at 5% increments of the tensile strength and the specimen was observed. After the transverse crack occurred, the load was applied at 10% increments of the tensile strength and it was observed. The observable or significant relaxation effects by removing the specimen were not found. 

### 3.3. Tensile Fatigue Testes

Tensile fatigue tests were carried out under load control using a hydraulic fatigue testing machine (Shimadzu Corp., Kyoto, Japan). All tests were run at a stress ratio of *R* = 0.1 and a frequency *f* = 5 or 100 Hz. The fatigue test conditions are listed in Table 3. *σ*_max_ is the maximum stress of the applied loading. As demonstrated in previous studies [32,37], the effect of the frequency on damage growth is weak for these test conditions. The fatigue tests were interrupted at arbitrary loading cycles based on *N* = 1.0 × 10*^n^*, 2.0 × 10*^n^*, 5.0 × 10*^n^* cycles as a standard, and the initiation and multiplication behavior of transverse cracks formed in the specimens was evaluated by ex-situ observation employing soft X-ray photography and optical microscopy, like the static tensile tests. For detailed observation of the transverse crack formation, atomic force microscopy (AFM, Bruker Corp., Billerica, MA, USA) and scanning electron microscopy (SEM, Hitachi, Ltd., Tokyo, Japan) were used. 

## 4. Damage Observation

### 4.1. Damage Growth Behavior Observed Employing Soft-X ray Photography

**Figure 2:**
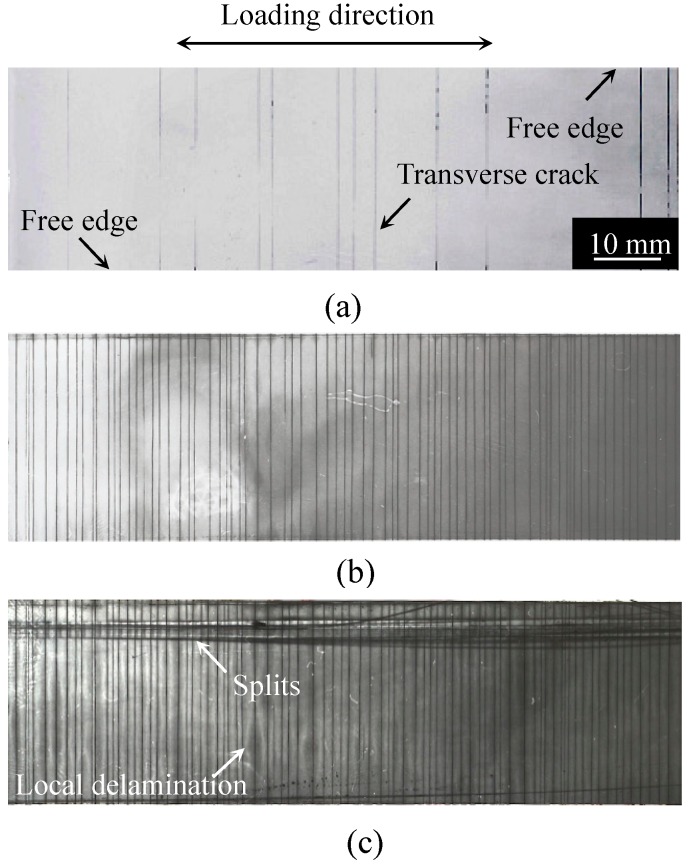
Ex-situ observation of the internal damage growth of [0/90_2_]_S_ laminate formed from the T800H/3631 prepreg under an applied stress level of *σ*_max_/*σ*_ti_ = 0.7: (**a**) *N* = 5.0 × 10^3^ cycles, (**b**) *N* = 1.0 × 10^5^ cycles, and (**c**) *N* = 1.0 × 10^6^ cycles [33].

**Figure 3:**
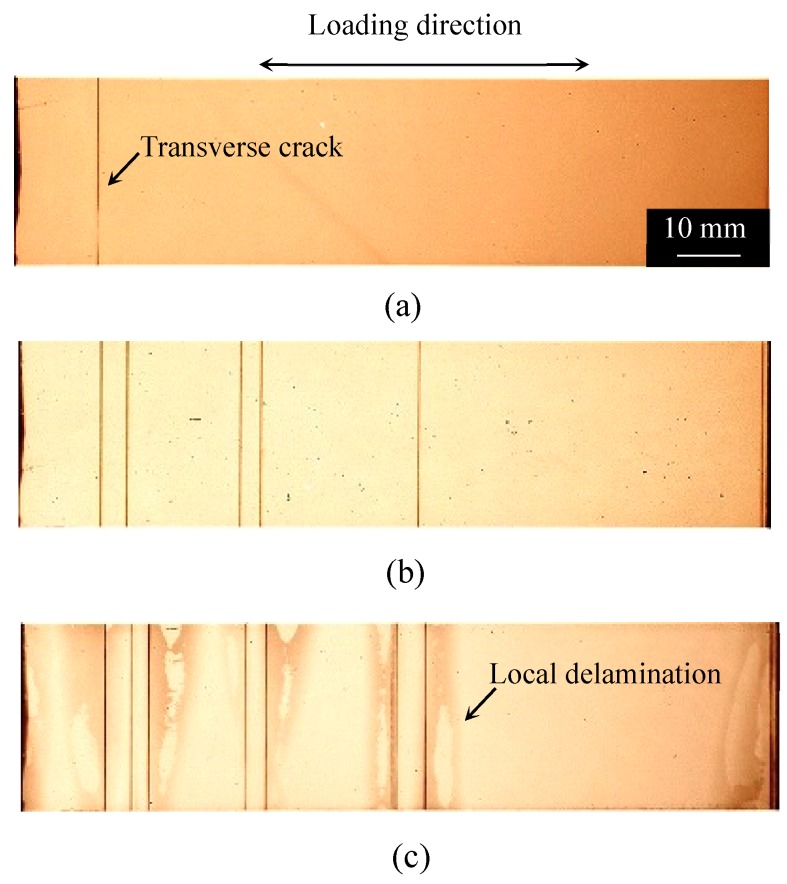
Ex-situ observation of the internal damage growth of [0_2_/90_12_]_S_ laminate formed from the T800S/2592 prepreg under an applied stress level of *σ*_max_/*σ*_ti_ = 1.0: (**a**) *N* = 1.0 × 10^2^ cycles, (**b**) *N* = 1.0 × 10^4^ cycles, and (**c**) *N* = 5.0 × 10^4^ cycles [34].

**Figure 4:**
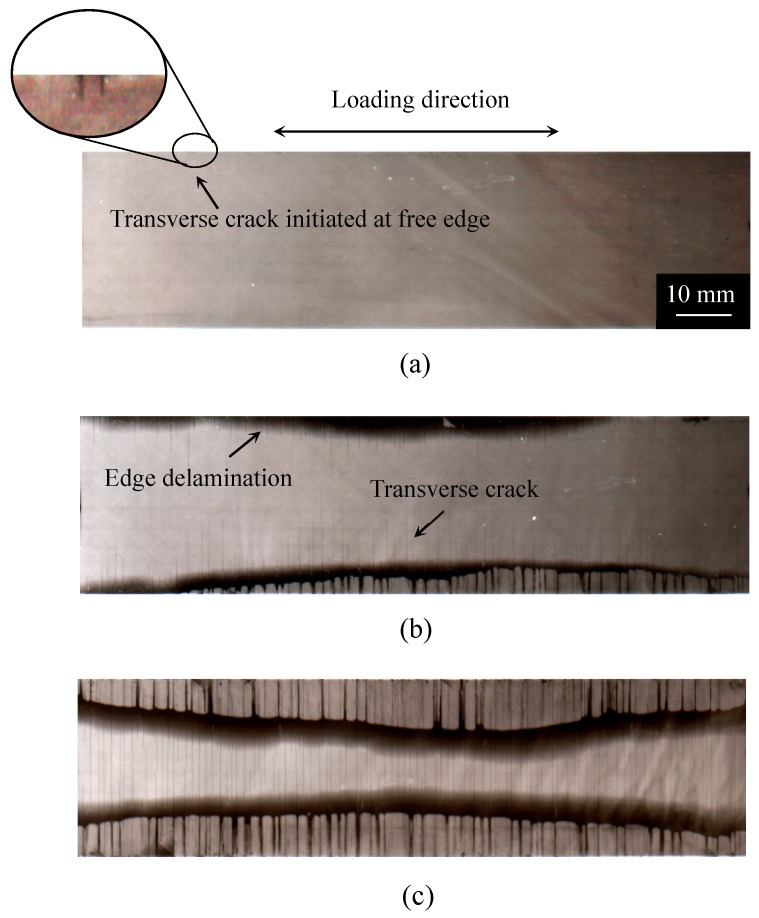
Ex-situ observation of the internal damage growth of [45/0/–45/90]_s_ laminate formed from the T800H/3631 prepreg under an applied stress level of *σ*_max_/*σ*_ti_ = 1.09: (**a**) *N* = 5.0 × 10^0^ cycles, (**b**) *N* = 5.0 × 10^2^ cycles, and (**c**) *N* = 5.0 × 10^3^ cycles [36] (with permission from Elsevier).

Figure 2, Figure 3 and Figure 4 show the damage growth behavior observed employing soft X-ray photography in the cross-ply [0/90_2_]_s_ laminate formed from the T800H/3631 prepreg, the cross-ply [0_2_/90_12_]_s_ laminate formed from the T800S/2592 prepreg, and the quasi-isotropic [45/0/–45/90]_s_ laminates formed from the T800H/3631 prepreg, respectively. In the cross-ply [0/90_2_]_s_ laminates of Figure 2, the transverse cracks that formed at the specimen edges rapidly grew in the width direction and multiplied in the specimen with an increasing number of loading cycles. Finally, local delamination originating from the transverse cracks and splitting occurred. In the cross-ply [0_2_/90_12_]_s_ laminates of Figure 3, the transverse cracks that formed at the specimen edges rapidly grew in the width direction. Unlike the case in Figure 2, local delamination propagated in the longitudinal direction after some transverse cracks formed. The transverse crack density was, therefore, much lower than that in Figure 2. The local delamination initiated and propagated more easily than in the thinner cross-ply laminates because a larger interlaminar shear stress developed at the transverse crack tip in the thick cross-ply laminates. The damage growth behavior of the cross-ply [0/90_6_]_s_ laminates formed from the same prepreg of the cross-ply [0_2_/90_12_]_s_ laminates was similar to the cross-ply [0/90_2_]_s_ laminates as shown in Figure 2 [34,35]. However, in the quasi-isotropic laminates shown in Figure 4, short transverse cracks initiated and saturated at the specimen edge, and then propagated in the width direction. Afterwards, delamination initiated in the interlaminar areas of the 90°/−45° plies at the specimen edges because of the stress concentration at the transverse crack tip. After the delamination grew in the longitudinal direction at the specimen edges, it propagated in the specimen width direction. The short transverse cracks and edge delamination were due to the free-edge effect, which is a stress singularity at the free edge caused by the difference in the Poisson’s ratios of each lamina. The damage growth behavior greatly differed depending on the laminate configuration. Since the initiation and growth of transverse cracks are greatly affected by the free edge effect, it is important to consider the influence of the free edge effect.

### 4.2. Observation of Transverse Crack Initiation and Growth

**Figure 5:**
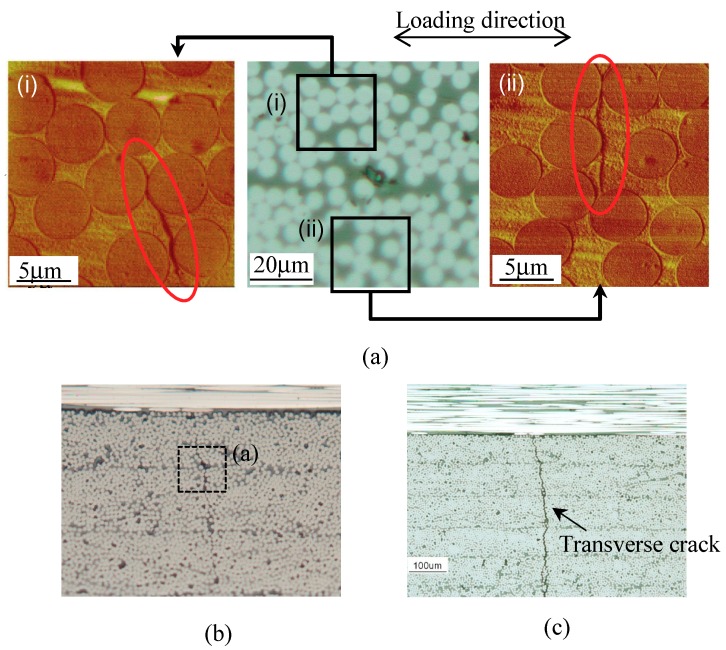
Observation of a micro crack growing from initial defects in 90° layers of cross-ply [0_2_/90_12_]_s_ laminates formed from the T800S/2592, *V*_f_ = 68% prepreg at an applied stress level of *σ*_max_/*σ*_ti_ = 0.7: (**a**) *N* = 0 cycles, (**b**) *N* = 1.0 × 10^4^ cycles, and (**c**) *N* = 5.0 × 10^5^ cycles.

Figure 5 shows the growth process of a micro crack originating from the initial defects in the cross-ply [0_2_/90_12_]_s_ laminates formed from the T800S/2592, *V*_f_ = 68% prepreg. The left and right images of the upper part of Figure 5 were observed using AFM. The other photographs in Figure 5 were observed using optical microscopy. The AFM samples were prepared by the following procedure. First, a replica film, acetyl cellulose film, which was applied with methyl acetate and attached to the edge surface of the specimen, and the micro damage of the specimen was transferred. The transferred film was attached to a sample stage and the surface was observed. The transfer resolution was approximately 100 Å. The initial defects are shown in Figure 5a. In addition, initial defects, with lengths of approximately 10 μm and depths of approximately 100 nm, were observed employing AFM. The micro cracks propagated in the thickness direction from these initial defects. The micro cracks were observed at *N* = 1.0 × 10^4^ cycles using an optical microscope. The micro cracks generally initiated near the 0°/90° interlaminar area and propagated to the opposite side.

**Figure 6:**
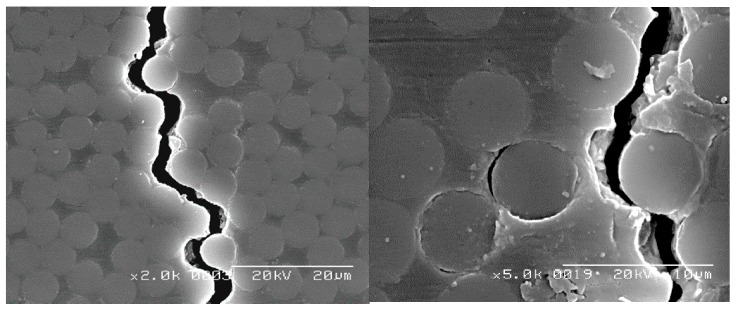
Observation of a micro crack growing along the interface between a fiber and the matrix in 90° layers of cross-ply [0/90_6_]_s_ laminates formed from the T800S/2592, *V*_f_ = 68% prepreg at *N* = 5.0 × 10^5^ cycles under an applied stress level of *σ*_max_/*σ*_ti_ = 0.8.

Figure 6 presents SEM photographs of a micro crack in the cross-ply [0/90_6_]_s_ laminates formed from the T800S/2592, *V*_f_ = 68% prepreg. In the SEM observation, the specimen was removed from the fatigue testing machine, and the edge surface of the specimen was coated with a platinum thin film. Then, the edge surface was observed under the condition of 20 kV. The micro crack propagated along the interface between the fiber and matrix under fatigue loading, whereas it propagated in the matrix under static tensile loading. Similar behavior was observed in the material system used in this study. In this study, a crack that passed through the thickness direction of the 90° layers in the laminates and could be observed employing soft X-ray photography was defined as a transverse crack.

## 5. Prediction Results of Transverse Crack Initiation under Fatigue Loading

**Figure 7:**
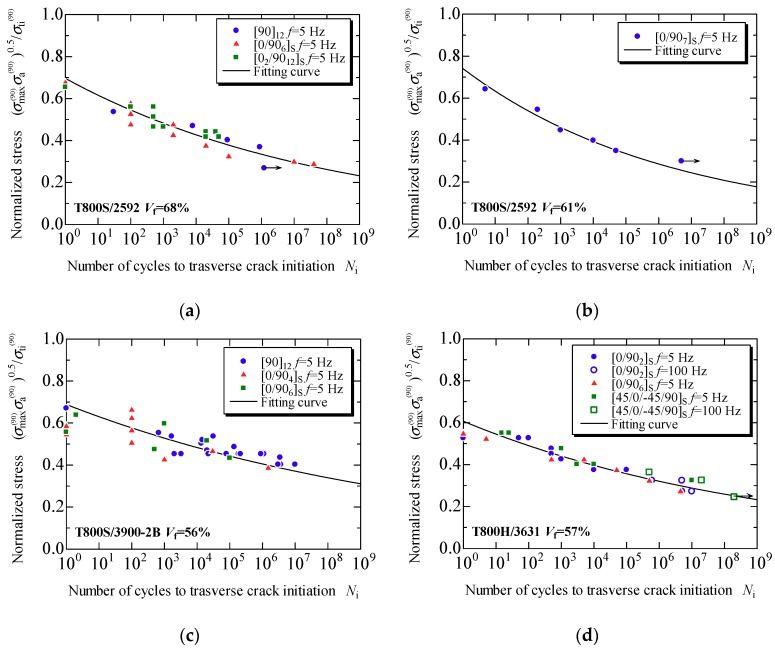
Relationship between the normalized applied stress obtained using the proposed model and the fatigue life to transverse crack initiation in specimens formed from each prepreg: (**a**) T800S/2592, *V*_f_ = 68%; (**b**) T800S/2592, *V*_f_ = 61%; (**c**) T800S/3900-2B, *V*_f_ = 56%; and (**d**) T800H/3631, *V*_f_ = 67%.

**Figure 8:**
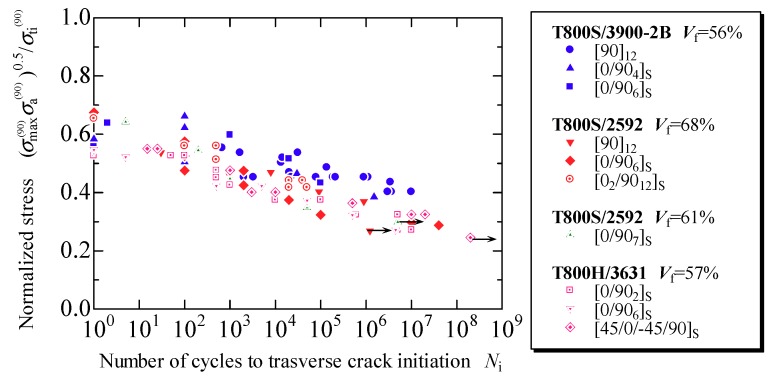
Relationship between the normalized applied stress obtained using the proposed model and the fatigue life to transverse crack initiation for all specimens.

Figure 7a–d presents the evaluation results obtained using the proposed model for the fatigue life prediction of the transverse crack initiation of laminates formed using each prepreg, T800S/2592 with *V*_f_ = 68% and 61%, T800S/3900-2B with *V*_f_ = 56%, and T800H/3631 with *V*_f_ = 57%, respectively. The plots in Figure 7 are the experimental data obtained from the fatigue tests using the laminates shown in Table 1. In the cross-ply and the quasi-isotropic laminates, the damage observation was repeatedly done, and the number of loading cycles in which a transverse crack was observed for the first time was measured. In the 90° unidirectional laminates, the number of cycles to the fracture was measured. The solid lines in the figure are the fitting curve obtained from Equation (11). The constants in Equation (11) for the prepregs of T800S/2592 with *V*_f_ = 68% and 61%, T800S/3900-2B with *V*_f_ = 56%, and T800H/3631 with *V*_f_ = 57% were *η* = 1.09 × 10^−3^ and *λ* = −18.8; *η* = 1.33 × 10^−2^ and *λ* = −14.5; *η* = 8.99 × 10^−5^ and *λ* = −25.4; and *η* = 2.64 × 10^−5^ and *λ* = −21.3, respectively. In this study, *γ* = 0.5, which corresponds to the Smith–Watson–Topper model [38], was used. The fatigue life of transverse crack initiation can be evaluated with a curve regardless of the laminate configuration. Figure 8 combines Figure 7a–d. Similar tendencies were observed for the transverse crack initiation with the various combinations of carbon fibers and epoxy resin used in this study; however, slight relative merits were observed among the prepregs.

**Figure 9:**
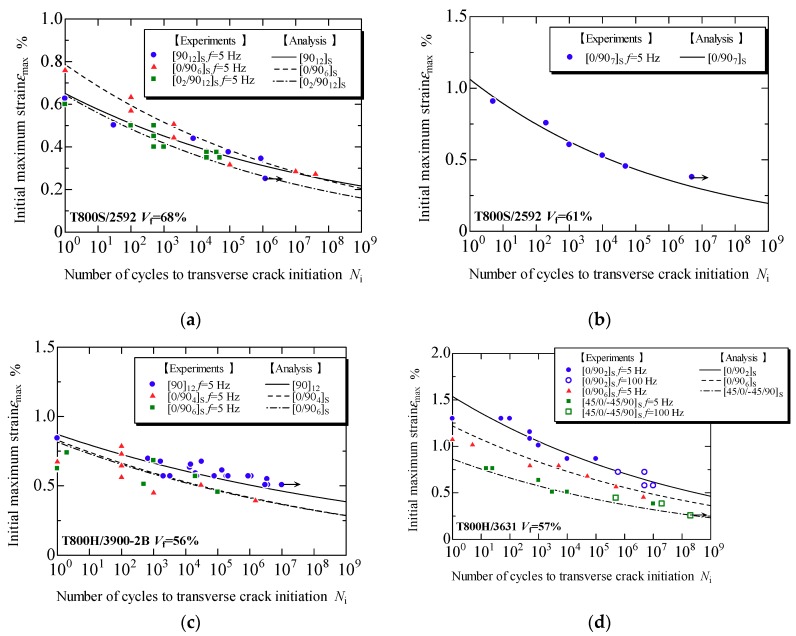
Relationship between the initial maximum strain and the fatigue life to transverse crack initiation in specimens formed from each prepreg: (**a**) T800S/2592, *V*_f_ = 68%; (**b**) T800S/2592, *V*_f_ = 61%; (**c**) T800S/3900-2B, *V*_f_ = 56%; and (**d**) T800H/3631, *V*_f_ = 67%.

Figure 9a–d shows the relationship between the initial maximum strain and the fatigue life to transverse crack initiation using the constants obtained from Figure 7. The initial maximum strain was calculated using Equation (7) because the values of strain are useful for the design of composite structures. The analytical results obtained using the proposed model are in good agreement with the experimental results. These findings indicate that the proposed model successfully considers the effects of the stress ratio, laminate thickness, and laminate configuration on transverse crack initiation under fatigue loading.

A tensile thermal residual stress was applied to the 90° layers in cross-ply and quasi-isotropic laminates at the test temperature. Thus, the stress ratio in the 90° layers, where the transverse crack initiates, varied depending on the load level applied to the specimen even if a mechanical stress with a stress ratio of *R* = 0.1 was applied. Nevertheless, the prediction results are in good agreement with the experimental results. From the results presented in Figure 9a, the fatigue life to the transverse crack initiation of [0/90_6_]_s_ laminates was approximately 30 times longer than that of [0_2_/90_12_]_s_ laminates despite the equivalent stress conditions of these laminates. These results indicate that the proposed model successfully considers the effect of the laminate thickness on the transverse crack initiation. In addition, the results in Figure 9a–c demonstrate that the fatigue life of the transverse crack initiation in cross-ply laminates can be predicted using the S–N curve of the 90° unidirectional laminates. The results in Figure 9d indicate that the fatigue life of the transverse crack initiation can be predicted for the quasi-isotropic laminates that cause the edge cracks as shown in Figure 4.

## 6. Discussion

### 6.1. Effect of Laminate Thickness

We consider the fatigue life of the transverse crack initiation of cross-ply [0/90_6_]_s_ laminates to be approximately 30 times longer than that of cross-ply [0_2_/90_12_]_s_ laminates, as observed in Figure 9a, because of the constraint effect of adjacent layers [26,39]. Figure 5 and Figure 6 demonstrate that a micro crack initiated near adjacent plies in the 90° layers and propagated to the opposite side in the 90° layers along the interface between the fiber and matrix under fatigue loading. During the micro crack propagation, the crack opening displacement of the micro crack in the 90° layers was smaller because of the restraint of the adjacent ply, the 0° ply, in the thinner laminates. Therefore, the energy release rate for the micro crack propagation is thought to decrease in the thinner laminates. Consequently, more cyclic loadings were required in the thinner laminates such that the micro cracks passed through the thickness direction in the 90° layers. However, the transverse crack in the quasi-isotropic laminates initiated at the low strain level even though the quasi-isotropic laminates had thin 90° plies. This initiation occurred because of the small constraint effect of −45° plies of adjacent layers and the free-edge effect. For the proposed model, the shape function of the laminates was erased by normalizing the energy release rate in Equation (10). It is thus thought that the transverse crack initiation can be evaluated regardless of the laminate thickness.

### 6.2. Free-Edge Effects

**Figure 10:**
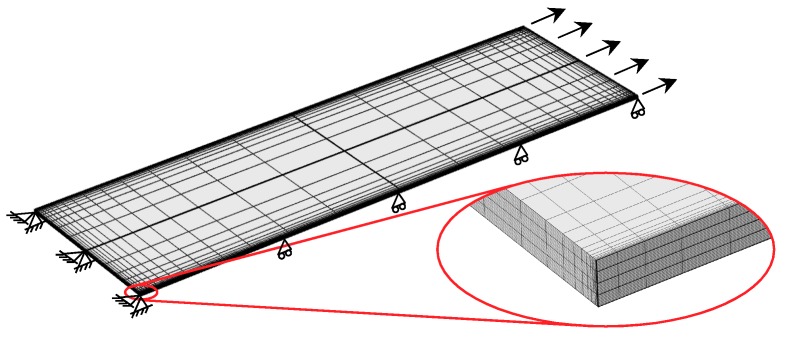
Finite element model of quasi-isotropic [45/0/–45/90]_s_ laminates.

**Figure 11:**
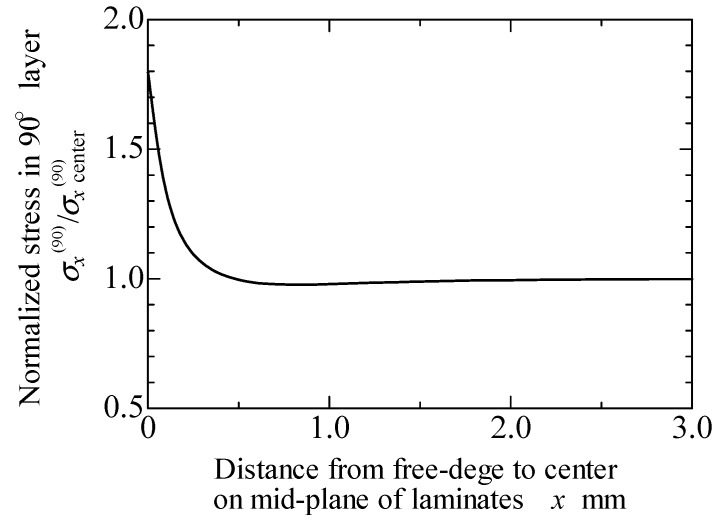
Stress singularity near the free edge in 90° layers of quasi-isotropic laminates calculated through finite element analysis.

**Figure 12:**
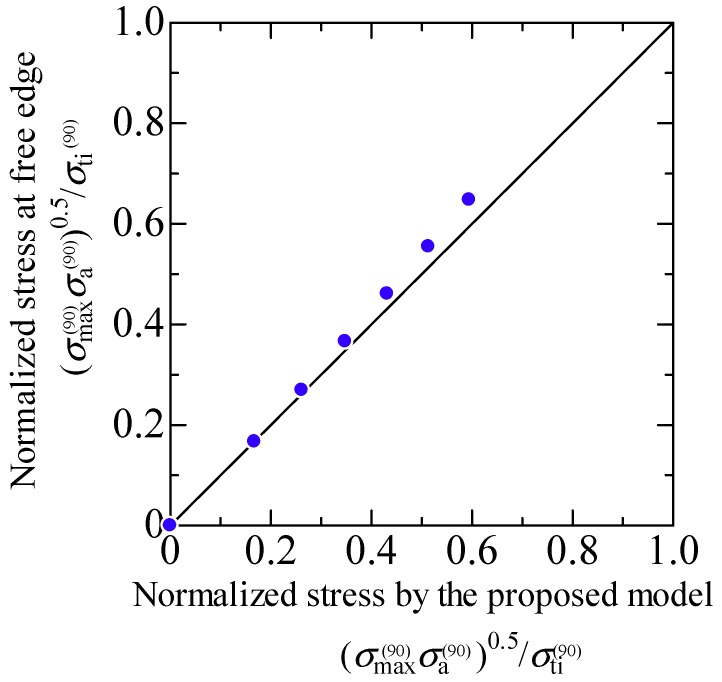
Comparison of the normalized stress at the free edge calculated through finite element analysis and the normalized stress calculated using the proposed model.

As mentioned above, the stress singularity due to the free-edge effect affected the initiation of transverse cracks in the quasi-isotropic laminates. As observed in Figure 2, Figure 3 and Figure 4, the transverse crack in the cross-ply laminates passed through the width direction immediately after it formed at the specimen edge, whereas the transverse crack in the quasi-isotropic laminates first multiplied at the specimen edge and then gradually propagated in the width direction. The model proposed in this study did not consider the stress singularity at the specimen edges. Nevertheless, the analytical results are in good agreement with the experimental results. Figure 10 presents a half model of the quasi-isotropic laminates produced using the commercial finite element code, COMSOL (a product name). A quadratic hexahedral element was used in the model. The number of elements was 19,200, and the minimum element size was 2.5 μm. Tensile loading was applied in the longitudinal direction. The edge surfaces of the specimen were well polished, and the surface roughness was *R*_a_ = 0.5 μm or less. The layers of the laminate were assumed as homogeneous orthotropic materials, and the stress was calculated by linear elastic analysis. It is thought that the stress at the edge surface of the FEM model is estimated higher than the actual. Figure 11 shows the relationship between the normalized normal stress distribution applied in the 90° layers of the laminates in the loading direction and the distance from the free edge when the loading corresponding to the stress of the transverse crack initiation under static tensile loading, *σ*_ti_, was applied to the model. The stress at the free edges was 1.8 times that inside. Figure 12 compares the normalized stress at the free edge, calculated through finite element analysis, with that calculated using the law of mixtures presented as Equation (7). The effect of the stress singularity at the free edge was canceled out by normalizing it with *σ*_ti_^(90)^. It is thus thought that the initiation of a transverse crack can be evaluated using Equation (11) for quasi-isotropic laminates. 

## 7. Conclusions

A model was proposed to predict transverse crack initiation in cross-ply and quasi-isotropic CFRP laminates under fatigue loading based on the Walker model. It was shown that the fatigue life can be predicted with three independent constants of the proposed model, *λ*, *η*, and *γ*, which are the parameters with physical meanings related to the fatigue strength reduction in the transverse crack initiation, the normalized fatigue strength in the transverse crack initiation at *N* = 1 cycle, and the contribution of stress amplitude on the fatigue life to the transverse crack initiation, respectively. The analytical results obtained using the proposed model were in good agreement with the experimental results, and the usefulness of the model was shown to predict the fatigue life to transverse crack initiation in cross-ply and quasi-isotropic CFRP laminates under tensile fatigue loading.

## Figures and Tables

**Table 1:** Specimen dimensions and laminate configurations.

Prepreg	*V*_f_ [%]	Laminate Configuration	Cure Temp. [K]	*L*_O_ [mm]	*L*_G_ [mm]	*W* [mm]	*t* [mm]	*L*_T1_ [mm]	*L*_T2_ [mm]
T800S/2592	68	[90]_12_	408	240	130	25	0.98	40	55
T800S/2592	68	[0/90_6_]_S_	408	230	120	30	1.1	40	55
T800S/2592	68	[0_2_/90_12_]_S_	408	230	120	30	2.2	40	55
T800S/2592	61	[0/90_7_]_S_	408	230	120	30	1.8	40	55
T800S/3900-2B	56	[90]_12_	453	200	100	25	2.3	50 *	50 *
T800S/3900-2B	56	[0/90_4_]_S_	453	230	120	30	1.9	40	55
T800S/3900-2B	56	[0/90_6_]_S_	453	230	120	30	2.7	40	55
T800H/3631	57	[0/90_2_]_S_	453	210	120	30	0.86	30	45
T800H/3631	57	[0/90_6_]_S_	453	210	120	30	2.0	30	45
T800H/3631	57	[45/0/–45/90]_S_	453	210	100	30	1.1	40	55

* Emery paper tabs were used.

**Table 2:** Mechanical properties of each prepreg.

Mechanical Properties		Unit	T800S/2592 (*V*_f_ = 68%)	T800S/2592 (*V*_f_ = 61%)	T800S/3900-2B (*V*_f_ = 56%)	T800H/3631 (*V*_f_ = 57%)
Longitudinal Young’s modulus	*E*_L_	[GPa]	186	164	146	165
Transverse Young’s modulus	*E*_T_	[GPa]	10.3	9.2	8.1	8.0
In-plane shear modulus	*G*_LT_	[GPa]	5.3	4.5	4.2	4.2
Out-of-plane shear modulus *	*G*_TT_	[GPa]	3.5	3.1	2.7	2.7
In-plane Poisson’s ratio	*ν*_LT_		0.33	0.35	0.35	0.32
Out-of-plane Poisson’s ratio **	*ν*_TT_		0.49	0.49	0.49	0.49
Longitudinal thermal expansion coefficient	*α*_L_	[×10^−6^/K]	0.35	0	0.2	0.1
Transverse thermal expansion coefficient	*α*_T_	[×10^−6^/K]	28	25	34	36

* *G*_TT_ was calculated from the equation, *G*_TT_ = *E*_T_/{2(1 + *ν*_TT_)}, ** Assumed value.

**Table 3:** Fatigue test conditions.

Prepreg	*V*_f_ [%]	Laminate Configuration	*σ*_max_/*σ*_ti_	*f* [Hz]	*R*	*σ*_ti_ [MPa]
T800S/2592	68	[90]_12_	0.40–1.00 *	5	0.1	64.6 *
T800S/2592	68	[0/90_6_]_S_	0.43–1.20	5	0.1	224
T800S/2592	68	[0_2_/90_12_]_S_	0.71–1.21	5	0.1	175
T800S/2592	61	[0/90_7_]_S_	0.45–1.07	5	0.1	242
T800S/3900-2B	56	[90]_12_	0.60–1.00 *	5	0.1	68.3 *
T800S/3900-2B	56	[0/90_4_]_S_	0.70–1.40	5	0.1	200
T800S/3900-2B	56	[0/90_6_]_S_	0.80–1.30	5	0.1	158
T800H/3631	57	[0/90_2_]_S_	0.60–0.90	5	0.1	871
			0.40–0.50	100		
T800H/3631	57	[0/90_6_]_S_	0.40–0.95	5	0.1	344
T800H/3631	57	[45/0/–45/90]_S_	0.55–1.09	5	0.1	426
			0.36–0.64	100		

* The stress of crack initiation for unidirectional [90]_12_ laminates was taken as the tensile strength σb and not σti.
